# Benefit of chemotherapy based on platinum with definitive radiotherapy in older patients with locally advanced esophageal squamous cell carcinoma

**DOI:** 10.1186/s13014-021-01931-1

**Published:** 2021-10-30

**Authors:** Haishan Wu, Yilin Yu, Qunhao Zheng, Tianxiu Liu, Yahua Wu, Zhiping Wang, Hongying Zheng, Lingyun Liu, Jiancheng Li

**Affiliations:** Fujian Medical University Cancer Hospital, Fujian Cancer Hospital, Fuzhou, 350014 China

**Keywords:** Esophageal squamous cell carcinoma, Chemoradiotherapy, Radiotherapy, Overall survival, Progression-free survival

## Abstract

**Objective:**

There is still no definitely therapeutic evidence of a beneficial effect of chemotherapy with radiotherapy for older patients with esophageal squamous cell carcinoma (ESCC). We aim to determine the influence of chemoradiotherapy (CRT) and radiotherapy (RT) alone in patients aged 65 years or older with locally advanced ESCC.

**Methods:**

We retrospectively analyzed 581 ESCC patients who underwent CRT and RT alone. Univariate and multivariate Cox regression analysis was used to analyze the impact of clinical factors on long‐term overall survival (OS) and progression-free survival (PFS). Finally, we compared the toxicity rates of these patients.

**Results:**

The median OS and PFS of the overall population were 23.2 months (2.0–162.6 months) and 18.6 months (1.1–159.6 months). Multivariate Cox regression analysis showed that chemotherapy (*p* < 0.05), tumor thickness (*p* < 0.01), and N stage (*p* < 0.05) were independent prognostic factors associated with both OS and PFS. In the chemotherapy subgroup, patients who received 2–8 cycles of chemotherapy had better OS than those who received 1 cycle (*p* = 0.015). The results also revealed that the CRT group has better OS and PFS than RT alone group for patients aged 65–74 years (both *p* < 0.01). However, for patients aged 75 years or older, there was no statistically significant difference between CRT and RT alone (both *p* > 0.05). Besides, higher staged ESCC has the inferior OS and PFS than lower staged ESCC for patients received RT alone and aged 65–74 years (both p < 0.05). Finally, there were significantly more severe hematologic toxicities in the CRT group than in those treated with RT alone in this study (*p* < 0.001).

**Conclusions:**

The present study suggested that CRT for locally advanced ESCC in patients aged 65 years or older had a significant benefit over RT alone in terms of OS and PFS. However, for patients aged 75 years or older, there was no statistically significant difference between CRT and RT alone. CRT should be performed with special attention in patients aged 75 years or older.

## Introduction

Esophageal carcinoma remains one of the most common cancers worldwide and is considered a serious malignant tumor in terms of prognosis and mortality rate. Every year, more than 550,000 new cases of esophageal carcinoma (EC) are diagnosed [1, 2]. It occurs mainly in the middle-aged and older individuals with a mean age of 67 years at diagnosis and about 30% of patients older than 75 years^3^–^5^. The number of older populations is increasing rapidly in the world [6, 7]. Due to the prolongation of life expectancy and aging of the global population increase, older patients with EC are increasing gradually over the recent decades. Cancer is the primary cause of death in older people. Therefore, this is sure to become a major challenge and an increasingly common social issue in the near future.

Despite many advances in surgical techniques, esophagectomy is associated with significant postoperative morbidity and mortality. The increased risk may be more vital in older patients due to a decreased physiological function, higher burden of comorbidities, and more inferior nutritional status [8, 9]. The older is often considered the limit for this type of surgery. There is a lack of evidence-based evidence regarding the appropriate treatment for the older population with locally advanced esophageal squamous cell carcinoma (ESCC). For patients who are either deemed medically inoperable or have unresectable tumors, the efficacy of definitive chemoradiotherapy (dCRT) has been proved in some randomized, controlled trials for EC patients^10^–^12^. However, older patients are under-represented in most randomized trials. Only a few studies have reported the efficacy and safety in elderly patients, thus questioning the feasibility of the results in the older population [13, 14]. Besides, most older patients are unlikely to be able to tolerate chemotherapy. Whether the treatment regime of younger patients can be applied to older patients is still controversial, and little data are available.

As the older population increases, it becomes even more critical to understand whether a standard approach should be used in the treatment of this challenging group of patients. To our knowledge, no high-level evidence specifically addressing the outcome of older patients treated by dCRT approaches has been published so far. Therefore, the standard therapy regimen for the older locally advanced ESCC patients has not reached a consensus yet. The aim of the present study was designed to retrospectively evaluate our hospital’s experience among the older patients with locally advanced ESCC who were treated with dCRT to better understand the feasibility, efficacy, and safety of this approach.

## Materials and methods

### Study population

A retrospective study was performed at the Fujian Provincial Cancer Hospital. All consecutive cases of 581 patients aged 65 or older with locally advanced esophageal squamous cell carcinoma (ESCC) who received definitive chemoradiotherapy (dCRT) from December 2007 to August 2020 were included. Patients’ inclusion criteria included: (A) aged 65 and older; (B) histopathologic proof of esophageal squamous cell carcinoma (ESCC); (C) Karnofsky Performance Status (KPS) ≥ 70 points; (D) no distinct metastasis or multiple primary diagnoses; (E) had not undergone esophagectomy; (F) no other major diseases (renal failure, liver failure and severe cardiovascular and cerebrovascular diseases). All the patients were diagnosed with locally advanced ESCC (stage II–IVA). The blood biochemical data was collected within three days before therapy. The tumor length was defined by barium esophagography and/or esophagoscopy, and tumor diameter by computed tomography (CT) scan and/or endoscopic ultrasound. For clinical staging, the eighth tumor-node-metastasis (TNM) edition was performed in all patients for esophageal carcinoma (EC). This study was carried out according to the declaration of Helsinki principles and approved by our Institutional Ethics Board (K2021-086-01).

### Radiotherapy

For treatment planning, the tumor volume was delineated using all available diagnostic information (barium esophagography, esophagoscopy, endoscopic ultrasound, CT, and positron emission tomography (PET)-CT). All plans were completed in the treatment plan system (Philips Pinnacle, USA). CT-based radiation planning and intensity modulated radiotherapy (IMRT) were used in the patients. All patients were treated with a total dose of 50–70 Gy (1.8–2 Gy per fraction, 1 fraction per day, and 5 days per week). The radiation parameters were as follows: (A) energy, 6 MV X-rays linear accelerator; (B) The gross tumor volume (GTV), including primary tumor and involved lymph nodes; (C) The clinical target volume (CTV) comprised GTV with at least 3 cm superior and inferior margins and at least 0.5 cm lateral margins; (D) The planned target volume (PTV), including a minimum of 0.5–1 cm surrounding the CTV. The dose and volume constraints for normal tissues were as follows: to the spinal cord, < 45 Gy; to the heart, V40 < 40%; And for the Bi-lung, average dose (MLD) ≤ 18 Gy, V5 ≤ 65%, V20 ≤ 30%.

### Chemotherapy

All of the 581 eligible patients had received 0–8 courses of concurrent or sequential chemotherapy. We used antiemetic drugs to prevent vomiting in the course of chemotherapy. The chemotherapy regimens were based on platinum, including (A) docetaxel 75 mg/m2 d1 or paclitaxel 135 mg/m2 d1 + nedaplatin 75 mg/m2 d2 or cisplatin 75 mg/m2 d2 or lobaplatin 50 mg d2 or carboplatin AuC2 d2; (B) 5-fluorouracil (5-FU) 700–1000 mg/m2 d1-2 + cisplatin 75 mg/m2 d2. Before and after every cycle of chemotherapy, complete blood biochemical data was obtained. Once severe toxicity happened, the chemotherapy dose would be adjusted in the next cycle.

### Toxicity

Toxicity was graded according to the Radiation Therapy Oncology Group (RTOG)/European Organization for Research and Treatment of Cancer radiation morbidity score system (EORTC). The grade of toxicity was scored retrospectively based on the medical records. An adverse effect at more than 3 months after completion of radiotherapy was defined as late toxicity. The toxicity was recorded as a maximum grade at any time during the treatment or follow-up period. Once severe radiation esophagitis/ pneumonitis/ hematologic toxicity appeared, both chemotherapy and radiotherapy would stop until recovery.

### Definition of the nutritional index and inflammatory index

The serum albumin level (g/L) + 5 multiplied by the absolute lymphocytes count to calculate the prognostic nutrition index (PNI). The absolute neutrophils count divided by the absolute lymphocytes count to calculate the neutrophils-lymphocytes ratio (NLR). The absolute platelets count divided by the absolute lymphocytes count was used to calculate the platelets-lymphocytes ratio (PLR). The absolute lymphocytes count divided by the absolute monocytes count to calculate the lymphocytes-monocytes ratio (LMR). Finally, the absolute platelets count multiplied by NLR to calculate the systemic immune-inflammation index (SII) [15].

### Evaluation methods and follow-up

All patients were followed up to detect survival status and disease progression every 3 months during the first years, every 6 months in the following 2 years, and once a year later until the end of the study. Follow-up involved physical examination, blood tests, biochemistry, tumor markers, upper gastrointestinal endoscopy, barium esophagography, CT, or PET-CT. Information about survival status and disease progression was updated until April 2021. The endpoint of the study was overall survival (OS) and progression-free survival (PFS). The OS is defined as the period from pathological diagnosis to death or the last follow-up. The PFS is defined as the period from pathological diagnosis to tumor progression, death, or last follow-up. The follow-up information came from the patient's clinical charts and / or telephone interviews. The median duration of follow-up was 23.2 months (range, 2.0 to 162.6 months).

### Statistical analysis

All statistical analyses were performed using R software (version 4.0.2) and SPSS software (version 26.0). The optimal cutoff values of radiotherapy (RT) dose, tumor length, tumor thickness, PNI, NLR, PLR, LMR, and SII are calculated using the X-tile application (https://medicine.yale.edu/lab/rimm/research/software/). Categorical data were compared by the Chi-square test or the Fisher exact test. Continuous variables were compared using the Mann–Whitney *U* test. The survival curve was drawn using the Kaplan–Meier method. The Cox regression model was used for univariate and multivariate analysis. In univariate analysis, all factors with *p* < 0.05 were involved in multivariate analysis to determine independent prognostic factors. All analyses were two-sided, and a *p* value < 0.05 was considered to be statistically significant.

## Results

### Patient stratification

The baseline characteristics of our study patients are shown in Table 1. A total of 581 patients underwent definitive chemoradiotherapy (dCRT), of whom 317 (54.6%) received chemotherapy and 264 (45.4%) did not. Only 101 (17.4%) patients received RT dose ≤ 58.8 Gy, and 480 patients received > 58.8 Gy. There were 316 (54.6%) patients aged 65–74 years and 265 (45.4%) aged 75 years or older. The median age of the patients was 74 years old (range, 65–90 years old). Overall, 373 (64.2%) were male, 148 (25.5%) were stage II, 180 (31%) were stage III, and 253 (43.5%) were stage IVA. Those treated with chemotherapy tended to be younger, were more likely to have stage IVA. The optimal cutoff value for RT dose, tumor length, tumor thickness, PNI, NLR, PLR, LMR, and SII was calculated to be 58.8, 5.8, 1.2, 41.7, 4.44, 180, 3.73, and 918, respectively.

**Table 1:** Patients’ characteristics of 581 locally advanced ESCC patients and patients’ clinicopathological characteristics according to chemotherapy

Clinicopathologic variable		Total(N)	CRT (n = 317)	RT (n = 264)	*p* value
Age (years)					< 0.001
	65–74	316 (54.4%)	245 (42.2%)	71 (12.2%)	
	≥ 75	265 (45.6%)	72 (12.4%)	193 (33.2%)	
Gender					0.005
	Male	373 (64.2%)	220 (37.9%)	153 (26.3%)	
	Female	208 (35.8%)	97 (16.7%)	111 (19.1%)	
Weight loss					0.023
	Yes	281 (48.4%)	167 (28.7%)	114 (19.6%)	
	No	300 (51.6%)	150 (25.8%)	150 (25.8%)	
RT dose (Gy)					0.567
	≤ 58.8	101 (17.4%)	52 (9.0%)	49 (8.4%)	
	> 58.8	480 (82.6%)	265 (45.6%)	215 (37.0%)	
Tumor location					0.118
	Cervical	37 (6.4%)	26 (4.5%)	11 (1.9%)	
	Upper thoracic	133 (22.9%)	78 (13.4%)	55 (9.5%)	
	Middle thoracic	338 (58.2%)	176 (30.3%)	162 (27.9%)	
	Lower thoracic	73 (12.6%)	37 (6.4%)	36 (6.2%)	
Tumor length (cm)					0.010
	≤ 5.8	344 (59.2%)	172 (29.6%)	172 (29.6%)	
	> 5.8	237 (40.8%)	145 (25.0%)	92 (15.8%)	
Tumor thickness (cm)					0.989
	≤ 1.2	232 (39.9%)	126 (21.7%)	106 (18.2%)	
	> 1.2	349 (60.1%)	191 (32.9%)	158 (27.2%)	
T stage					0.005
	T2	41 (7.1%)	16 (2.8%)	25 (4.3%)	
	T3	299 (51.5%)	152 (26.2%)	147 (25.3%)	
	T4	241 (41.5%)	149 (25.6%)	92 (15.8%)	
N stage					0.030
	N0	190 (32.7%)	91 (15.7%)	99 (17.0%)	
	N1	259 (44.6%)	143 (24.6%)	116 (20.0%)	
	N2	111 (19.1%)	67 (11.5%)	44 (7.6%)	
	N3	21 (3.6%)	16 (2.8%)	5 (0.9%)	
TNM stage					0.001
	Stage II	148 (25.5%)	63 (10.8%)	85 (14.6%)	
	Stage III	180 (31.0%)	96 (16.5%)	84 (14.5%)	
	Stage IVA	253 (43.5%)	158 (27.2%)	95 (16.4%)	
Year of diagnosis					0.059
	2007–2017	404 (69.5%)	210 (36.1%)	194 (33.4%)	
	2018–2020	177 (30.5%)	107 (18.4%)	70 (12.0%)	
PNI					0.048
	≤ 41.7	88 (15.1%)	39 (6.7%)	49 (8.4%)	
	> 41.7	493 (84.9%)	278 (47.8%)	215 (37.0%)	
NLR					0.025
	≤ 4.44	517 (89.0%)	291 (50.1%)	226 (38.9%)	
	> 4.44	64 (11.0%)	26 (4.5%)	38 (6.5%)	
PLR					0.278
	≤ 180	451 (77.6%)	252 (43.4%)	199 (34.3%)	
	> 180	130 (22.4%)	65 (11.2%)	65 (11.2%)	
LMR					0.426
	≤ 3.73	246 (42.3%)	129 (22.2%)	117 (20.1%)	
	> 3.73	335 (57.7%)	188 (32.4%)	147 (25.3%)	
SII					0.149
	≤ 918	482 (83.0%)	270 (46.5%)	212 (36.5%)	
	> 918	99 (17.0%)	47 (8.1%)	52 (9.0%)	

ESCC, esophageal squamous cell carcinoma; CRT, chemoradiotherapy; RT, radiotherapy; T, tumor; N, node; TNM, tumor-node-metastasis; PNI, prognostic-nutrition index; NLR, neutrophils-lymphocytes ratio; PLR, platelets-lymphocytes ratio; LMR, lymphocytes-monocytes ratio; SII, systemic immune-inflammation index

### Overall survival in different age groups

The median overall survival (OS) of the overall population was 23.2 months (range, 2.0–162.6 months), and 1, 3, and 5 years OS rates were 79.3%, 43.7%, and 31.7%, respectively. For patients treated with chemoradiotherapy (CRT) and radiotherapy (RT) alone, the median OS was 25 months (range, 2.9–124.4 months) and 19.5 months (range, 2.0–162.6 months), respectively (*p* < 0.01). The 1, 3, and 5 years OS rates in patients treated with CRT were 83.3%, 48.5%, and 36.7%, respectively, while in patients treated with RT alone were 74.5%, 38.0%, and 25.7%, respectively.

### Progression-free survival in different age groups

The median progression-free survival (PFS) of the overall population was 18.6 months (range, 1.1–159.6 months), and 1, 3, and 5 years PFS rates were 67.7%, 37.8%, and 28.9%, respectively. For patients treated with chemoradiotherapy (CRT) and radiotherapy (RT) alone, the median PFS was 20.4 months (range, 1.5–124.4 months) and 15.8 months (range, 1.1–159.6 months), respectively (p < 0.01). The 1, 3, and 5 years PFS rates in patients treated with CRT were 73.8%, 42.9%, and 33.8%, respectively, while in patients treated with RT alone were 60.4%, 31.8%, and 23.2%, respectively.

### Univariate and multivariate survival analysis of OS and PFS in ESCC

**Fig. 1:**
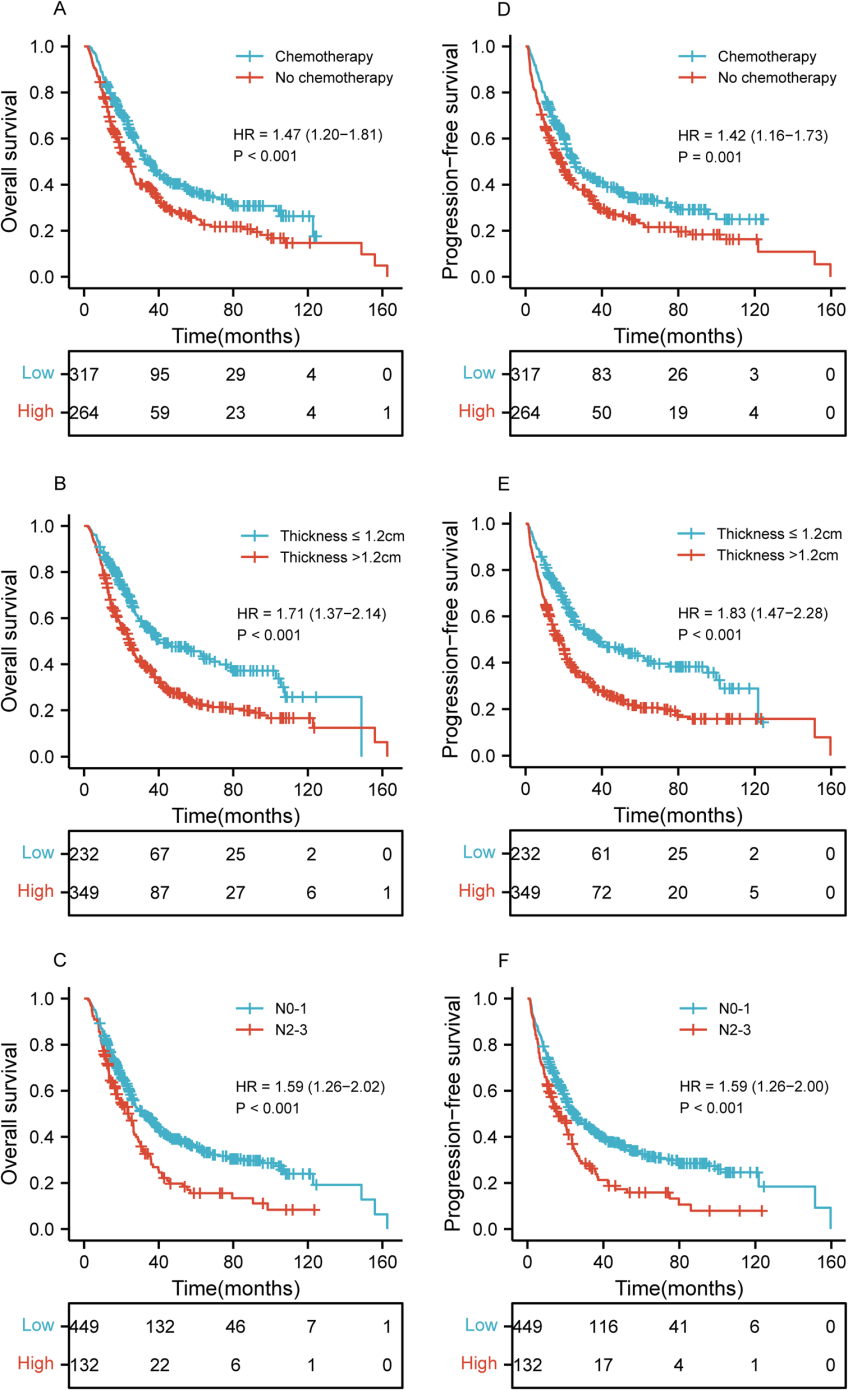
Kaplan–Meier curves of chemotherapy, tumor thickness, and N stage for the whole study population showing (**A**–**C**) overall survival (*p* < 0.001, *p* < 0.001, *p* < 0.001, respectively); (**D**–**F**) progression-free survival (*p* = 0.001, *p* < 0.001, *p* < 0.001, respectively). HR, hazard ratio; N, node

Univariate and multivariate Cox regression analyses for predictors of OS and PFS was shown in Tables 2 and 3. In Table 2, univariate analyses revealed that the age (*p* = 0.009), chemotherapy (*p* < 0.001), RT dose (*p* = 0.025), tumor length (*p* = 0.002), tumor thickness (*p* < 0.001), N stage (*p* < 0.001), TNM stage (*p* = 0.006), PNI (*p* < 0.001), NLR (*p* = 0.001), PLR (*p* = 0.001), LMR (*p* = 0.004), and SII (*p* < 0.001) were significant risk factors for a worse OS. On multivariate analysis, the chemotherapy (*p* = 0.007; HR, 1.405; 95% CI, 1.095–1.804), tumor thickness (*p* = 0.003; HR, 1.429; 95% CI, 1.126–1.813), and N stage (*p* = 0.011; HR, 1.385; 95% CI, 1.076–1.784) were independently associated with worse OS. In Table 3, univariate analyses demonstrated that the age (*p* = 0.013), chemotherapy (*p* = 0.001), tumor length (*p* = 0.002), tumor thickness (*p* < 0.001), N stage (*p* < 0.001), TNM stage (*p* = 0.002), Year of diagnosis (*p* = 0.028), PNI (*p* < 0.001), NLR (*p* = 0.001), PLR (*p* = 0.001), LMR (*p* = 0.001), and SII (*p* < 0.001) were significant risk factors for a worse PFS. On multivariate analysis, the chemotherapy (*p* = 0.022; HR, 1.328; 95% CI, 1.041–1.694), tumor thickness (*p* = 0.001; HR, 1.498; 95% CI, 1.184–1.894), and N stage (*p* = 0.006; HR, 1.418; 95% CI, 1.107–1.817) were independently associated with worse PFS. As shown in Fig. 1, there was a significant difference of OS and PFS in chemotherapy, tumor thickness, and N stage.

**Table 2:** Factors associated with overall survival: univariate and multivariate Cox proportional hazards models

Clinicopathologic parameters	Univariate analysis			Multivariate analysis		
	HR	95% CI	*p*	HR	95% CI	*p*
**Age (years)**						
≥ 75 vs. 64–75	1.314	1.069–1.614	0.009	1.156	0.900–1.484	0.256
**Gender**						
Male vs. Female	1.067	0.858–1. 326	0.560			
**Weight loss**						
Yes vs. No	0.918	0.747–1. 129	0.419			
**Chemotherapy**						
No vs. Yes	1.473	1.199–1. 811	< 0.001	1.405	1.095–1.804	0.007
**RT dose (Gy)**						
≤ 58.8 vs. > 58.8	1.356	1.039–1. 769	0.025	1.185	0.902–1.558	0.223
**Tumor location**						
Cervical/Upper vs. Middle/Lower	0.850	0.674–1. 073	0.171			
**Tumor length (cm)**						
> 5.8 vs. ≤ 5.8	1.389	1.129–1. 709	0.002	1.226	0.984–1.528	0.070
**Tumor thickness (cm)**						
> 1.2 vs. ≤ 1.2	1.711	1.369–2.137	< 0.001	1.429	1.126–1.813	0.003
**T stage**						
T4 vs. T2/T3	1.039	0.844–1. 279	0.716			
**N stage**						
N2/N3 vs. N0/N1	1.594	1.261–2.015	< 0.001	1.385	1.076–1.784	0.011
**TNM stage**						
Stage III/Stage IV vs. Stage II	1.415	1.102–1.817	0.006	1.217	0.924–1.602	0.162
**Year of diagnosis**						
2007–2017 vs. 2018–2020	1.153	0.873–1.522	0.316			
**PNI**						
≤ 41.7 vs. > 41.7	1.669	1.273–2.189	< 0.001	1.227	0.898–1.676	0.199
**NLR**						
> 4.44 vs. ≤ 4.44	1.672	1.226–2.281	0.001	1.135	0.771–1.669	0.521
**PLR**						
> 180 vs. ≤ 180	1.517	1.197–1.924	0.001	1.103	0.818–1.487	0.520
**LMR**						
≤ 3.73 vs. > 3.73	1.357	1.103–1.668	0.004	1.128	0.901–1.413	0.293
**SII**						
> 918 vs. ≤ 918	1.726	1.339–2.226	< 0.001	1.267	0.897–1.790	0.180

HR, hazard ratio; CI, confidence interval; RT, radiotherapy; T, tumor; N, node; TNM, tumor-node-metastasis; PNI, prognostic-nutrition index; NLR, neutrophils-lymphocytes ratio; PLR, platelets-lymphocytes ratio; LMR, lymphocytes-monocytes ratio; SII, systemic immune-inflammation index

**Table 3:** Factors associated with progression-free survival: univariate and multivariate Cox proportional hazards models

Clinicopathologic parameters	Univariate Analysis			Multivariate Analysis		
	HR	95% CI	*p*	HR	95% CI	*p*
**Age (years)**						
≥ 75 vs. 64–75	1.292	1.056–1.581	0.013	1.217	0.955–1.550	0.112
**Gender**						
Male vs. Female	1.158	0.935–1. 435	0.178			
**Weight loss**						
Yes vs. No	0.960	0.784–1. 175	0.692			
**Chemotherapy**						
No vs. Yes	1.416	1.157–1. 732	0.001	1.328	1.041–1.694	0.022
**RT dose (Gy)**						
≤ 58.8 vs. > 58.8	1.200	1.921–1. 564	0.176			
**Tumor location**						
Cervical/Upper vs. Middle/Lower	0.867	0.691–1. 088	0.219			
**Tumor length (cm)**						
> 5.8 vs. ≤ 5.8	1.377	1.124–1. 687	0.002	1.179	0.950–1.465	0.135
**Tumor thickness (cm)**						
> 1.2 vs. ≤ 1.2	1.830	1.472–2.276	< 0.001	1.498	1.184–1.894	0.001
**T stage**						
T4 vs. T2/T3	1.109	0.906–1. 359	0.316			
**N stage**						
N2/N3 vs. N0/N1	1.589	1.262–2.001	< 0.001	1.418	1.107–1.817	0.006
**TNM stage**						
Stage III/Stage IV vs. Stage II	1.485	1.161–1.900	0.002	1.286	0.981–1.687	0.069
**Year of diagnosis**						
2007–2017 vs. 2018–2020	1.345	1.033–1.752	0.028	1.293	0.986–1.695	0.063
**PNI**						
≤ 41.7 vs. > 41.7	1.682	1.290–2.193	< 0.001	1.237	0.912–1.677	0.172
**NLR**						
> 4.44 vs. ≤ 4.44	1.659	1.221–2.255	0.001	1.216	0.839–1.762	0.301
**PLR**						
> 180 vs. ≤ 180	1.507	1.193–1.902	0.001	1.140	0.849–1.529	0.384
**LMR**						
≤ 3.73 vs. > 3.73	1.417	1.157–1.734	0.001	1.161	0.932–1.447	0.184
**SII**						
> 918 vs. ≤ 918	1.715	1.335–2.203	< 0.001	1.246	0.890–1.744	0.200

HR, hazard ratio; CI, confidence interval; RT, radiotherapy; T, tumor; N, node; TNM, tumor-node-metastasis; PNI, prognostic-nutrition index; NLR, neutrophils-lymphocytes ratio; PLR, platelets-lymphocytes ratio; LMR, lymphocytes-monocytes ratio; SII, systemic immune-inflammation index

### Survival stratified by the cycle of chemotherapy

**Fig. 2:**
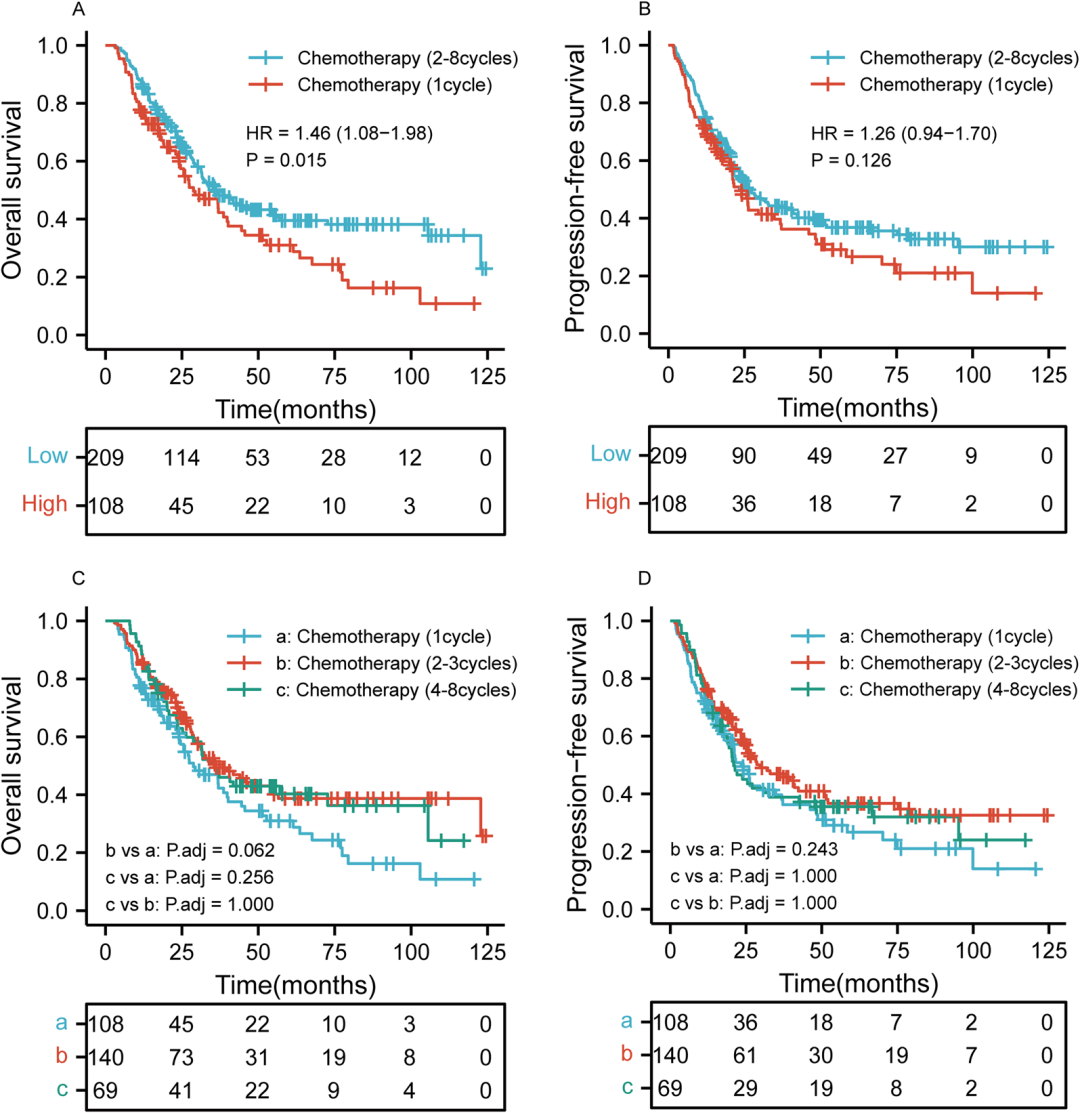
Kaplan–Meier curves according to cycle of chemotherapy categories among patients who received chemotherapy showing **A**–**B** overall survival (*p* = 0.015) and progression-free survival (*p* = 0.126) for 1 and 2–8 cycles of chemotherapy; **C**–**D** overall survival (*p* > 0.05) and progression-free survival (*p* > 0.05) for 1, 2–3, and 4–8 cycles of chemotherapy. HR, hazard ratio; Adj, adjust

We found that chemotherapy was an independent prognostic factor. In our study, 317 patients received 0–8 cycles of chemotherapy. In order to further analyze the relationship between the cycle of chemotherapy and OS and PFS, we classified the chemotherapy cycle into two categories and three categories. As shown in Fig. 2A–B, patients who received 2–8 cycles of chemotherapy had better OS than those who received 1 cycle of chemotherapy (*p* = 0.015), but PFS was not statistically significant (*p* = 0.126). However, Fig. 2C–D showed that there were no significant differences in OS and PFS among the patients who received 1, 2–3, and 4–8 cycles of chemotherapy (*p* > 0.05 for all).

### Survival stratified by age

**Fig. 3:**
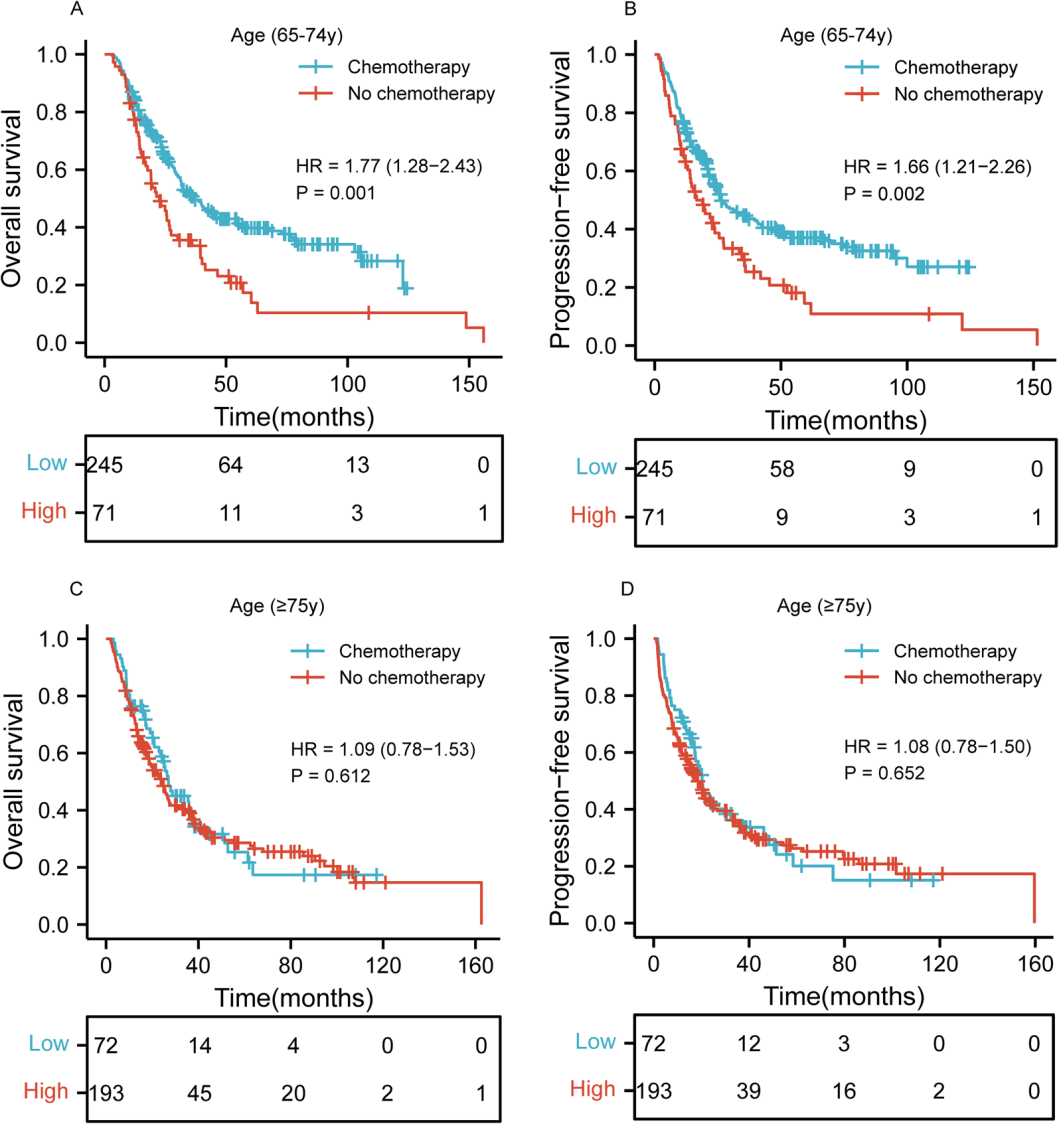
Kaplan–Meier curves according to age categories for the whole study population showing **A**–**B** overall survival (*p* = 0.001) and progression-free survival (*p* = 0.002) of patients aged 65–74 years; **C**–**D** overall survival (*p* = 0.612) and progression-free survival (*p* = 0.652) of patients aged 75 years or older. HR, hazard ratio

We further analyzed the effects of age and dCRT on OS and PFS. Figure 3A–B showed that CRT has the better OS and PFS than RT alone for patients aged 65–74 years (*p* = 0.001 and *p* = 0.002, respectively). However, for patients aged 75 years or older, there was no statistically significant difference between CRT and RT alone in Fig. 3C–D (*p* = 0.612 and *p* = 0.652, respectively).

### Survival stratified by age, applied therapy, and tumor stages

**Fig. 4:**
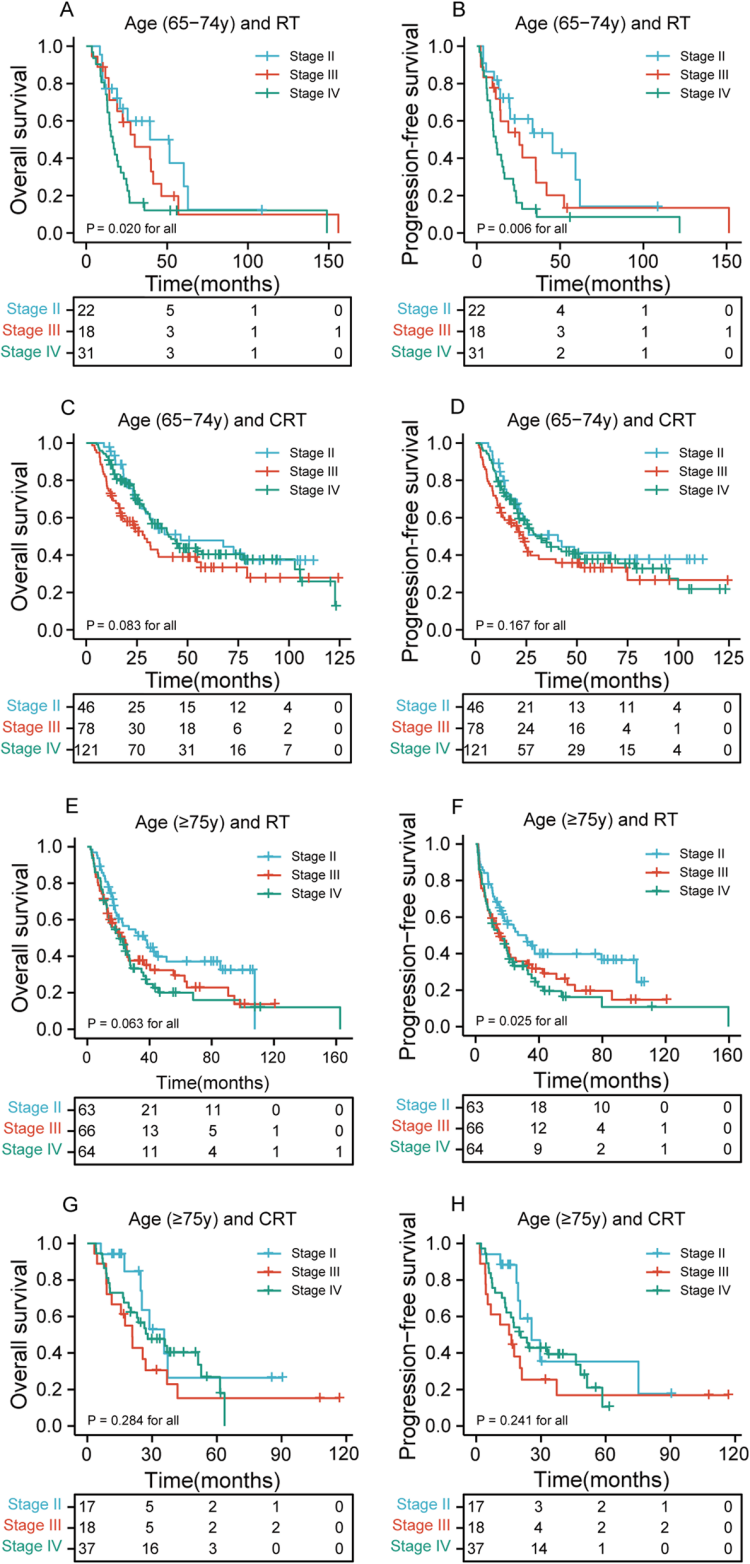
Kaplan–Meier curves according to age, applied therapy, and tumor stages categories for the whole study population showing **A**–**B** overall survival (*p* = 0.020 for all) and progression-free survival (*p* = 0.006 for all) of patients received RT alone and aged 65–74 years in different tumor stages; **C**–**D** overall survival (*p* = 0.083 for all) and progression-free survival (*p* = 0.167 for all) of patients received CRT and aged 65–74 years in different tumor stages; **E**–**F** overall survival (*p* = 0.063 for all) and progression-free survival (*p* = 0. 025 for all) of patients received RT alone and aged 75 years or older in different tumor stages; **G**–**H** overall survival (*p* = 0.284 for all) and progression-free survival (*p* = 0. 241 for all) of patients received CRT and aged 75 years or older in different tumor stages. RT, radiotherapy; CRT, chemoradiotherapy

We also analyzed the effects of age, applied therapy, and tumor stages on OS and PFS. Figure 4A–B showed that higher staged ESCC has the inferior OS and PFS than lower staged ESCC for patients received RT alone and aged 65–74 years (*p* = 0.020 for all and *p* = 0.006 for all, respectively). However, for patients received CRT, there was no statistically significant difference between higher staged ESCC and lower staged ESCC in Fig. 4C–D (*p* = 0.083 for all and *p* = 0.167 for all, respectively). Similarly, for patients received RT alone and aged 75 years or older, lower staged ESCC has the better PFS than higher staged ESCC, but not involve OS (Fig. 4E–F) (*p* = 0.063 for all and *p* = 0.025 for all, respectively). Finally, there was no statistically significant difference between lower staged ESCC and higher staged ESCC of patients received CRT in Fig. 4G–H (*p* = 0.284 for all and *p* = 0.241 for all, respectively).

### Toxicity

Grade 3–4 radiation esophagitis (RE) was identified in 6.9% (22/317) of the CRT group and 10.2% (27/264) of the RT alone group in Table 4 ((*p* = 0.158; OR, 1.528; 95% CI, 0.848–2.751)). Of the 581 patients, radiation pneumonitis (RP) occurred in 29 (9.1%) and 19 (7.1%) patients of CRT group and RT alone group ((*p* = 0.396; OR, 0.770; 95% CI, 0.421–1.408)). Hematologic toxicity grade ≥ 3 was observed in 51 (16.1%) and 7 (2.7%) of patients received CRT and RT alone, respectively ((*p* < 0.001; OR, 0.142; 95% CI, 0.063–0.319)).

**Table 4:** Toxicities of chemoradiotherapy and radiotherapy alone for all patients

Toxicity	CRT group (n = 317)	RT group (n = 264)	*p* value
**Radiation esophagitis**			0.158
Grade < 3	295	237	
Grade ≥ 3	22	27	
**Radiation pneumonitis**			0.396
No	288	245	
Yes	29	19	
**Hematologic toxicity**			< 0.001
Grade < 3	266	257	
Grade ≥ 3	51	7	

CRT, chemoradiotherapy; RT, radiotherapy

## Discussion

There is still no definitely therapeutic evidence of a beneficial effect of chemotherapy with radiotherapy for older patients with locally advanced esophageal squamous cell carcinoma (ESCC). Since the Radiation Therapy Oncology Group trial 85–01 indicated that the outcome of CRT was significantly better than that of RT alone for patients with esophageal cancer [10], CRT has been a standard treatment for esophageal carcinoma (EC). However, most of these studies typically included few patients aged 65 years or older. Besides, most elderly patients are unable to tolerate chemotherapy. Whether the therapeutic regimens from younger patients can be applied to elderly patients remains controversial, and data are scarcely available.

This study aimed to review our hospital's experience among the elderly patients with locally advanced ESCC who were treated with definitive chemoradiotherapy (dCRT) to better understand the efficacy of this approach in comparison to radiotherapy (RT) alone. To the best of our knowledge, our study has a relatively large sample of elderly patients aged 65 years or older for comparison. In the study, compared to the radiotherapy (RT) group, the chemoradiotherapy (CRT) group had significantly better overall survival (OS) and progression-free survival (PFS). Furthermore, multivariate Cox analysis showed that chemotherapy, tumor thickness, and N stage were regarded as independent prognostic factors that affect both the OS and PFS.

Several studies showed that CRT is an effective treatment in elderly patients with EC [16–19], which is similar to our multivariate analysis result. Whether CRT is effective and tolerable for elderly patients (> 65 years or ≥ 70 years) were analyzed by two retrospective studies which revealed significant results of CRT with a better survival benefit, which suggested that CRT can be effective and feasible for older patients [13, 17]. However, the sample size of these studies is relatively small. Additionally, most of these study populations are aged 75/80 years or older.

N stage and tumor thickness were found to be independent predictors affecting both OS and PFS in our study. As is known to us all, an increased number of positive lymph nodes and an increase in the lymph node classification were associated with a worse prognosis in esophageal carcinoma [20]. Although some studies demonstrated that tumor thickness is an independent prognostic factor for EC, there is still no consensus on the prognostic cutoff point of tumor thickness^21^, ^22^. Our study showed that the optimum cutoff value of tumor thickness was 1.2 cm, which is different from previous studies [21, 22]. The sample size and measuring method are the two crucial reasons for the inconsistency of the results.

In order to further analyze the relationship between the cycle of chemotherapy and OS and PFS, we classified the chemotherapy cycle into two categories and three categories. The result showed that patients who received 2–8 cycles of chemotherapy had better OS than those who received 1 cycle of chemotherapy (*p* = 0.015), but PFS was not statistically significant (*p* = 0.126). In addition, there were no significant differences in OS and PFS among the patients who received 1, 2–3, and 4–8 cycles of chemotherapy (*p* > 0.05 for all). It suggested that for patients receiving chemotherapy, 2 or more cycles of chemotherapy may be related to better OS.

Many studies have shown that age was not a risk factor of survival, which is similar to our result [13, 17, 23]. A previous study showed that life expectancy is over 10 years at 70 years of age [24]. Therefore, older patients with ESCC may benefit from curative treatment. Nonetheless, many physicians are hesitant to deliver curative CRT to elderly patients because of its severe toxicity in reality. In general, older patients are unlikely to tolerate chemotherapy [25, 26]. However, some studies suggested that CRT in older patients had survival benefits compared with RT alone [19, 27–29]. Conversely, Jingu et al. and Zhou et al. indicated that CRT for esophageal cancer in patients (≥ 80 years or ≥ 75 years) did not have statistically significant survival benefit from CRT compared with RT alone [30, 31]. In the present study, we further analyzed the effects of age and dCRT on OS and PFS. The results indicated that CRT has the better OS and PFS than RT alone for patients aged 65–74 years (both *p* < 0.01). Interestingly enough, for patients aged 75 years or older, there was no statistically significant difference between the CRT group and RT alone group (both *p* > 0.05). It suggested that CRT may have no benefit for elderly ESCC patients aged 75 years or older than RT alone.

As is known to all, higher staged cancers and milder types of therapy are identified as factors of poorer prognosis in EC. To clearly elaborate influence of the therapeutic effect on survival and course of disease, we also analyzed the effects of age, applied therapy, and tumor stages on OS and PFS. The result showed that higher staged ESCC has the inferior OS and PFS than lower staged ESCC for patients received RT alone and aged 65–74 years (*p* < 0.05). However, for patients received CRT, there was no statistically significant difference between higher staged ESCC and lower staged ESCC (*p* > 0.05). Similarly, for patients received RT alone and aged 75 years or older, lower staged ESCC has the better PFS than higher staged ESCC (*p* < 0.05).

So far, there is no evidence to suggest that advanced age is an independent contraindication for CRT in the retrospective studies. In our study, 317 (54.6%) patients received CRT compared to the RT alone (45.4%). The rate of grade ≥ 3 radiation esophagitis (RE) in the CRT group and the RT group had no statistical significance (*p* = 0.158). There was also no statistical difference in the incidence of radiation pneumonitis (RP) (*p* = 0.396). However, there was significantly more severe hematologic toxicity in the CRT group than in that treated with RT alone in this study (*p* < 0.001). In addition, several studies indicated that older age was found to be a risk factor for toxicity after dCRT for EC [32–35]. CRT should be conducted with special care for elderly patients. Our result showed that RT alone is regarded to be relatively safe. However, even RT alone requires special attention in elderly patients. Therefore, the potential benefit from CRT or RT should be carefully balanced against the risk of toxicity in elderly patients. Further research is needed to establish predictors that can identify risk factors for older patients and develop different selections for candidates who would benefit from different effective treatments.

Due to the retrospective nature, our study suffers from limitations. Firstly, this was a single institutional study, which may be subject to selection bias. Secondly, our study is limited to patients with ESCC and has no guiding significance for patients with other types of EC. Thirdly, RT dose and cycles of chemotherapy were different according to patients. Finally, as the study is, as clearly stated by us, of retrospective character, data is not powered towards selection of certain chemotherapy protocols in every aspect due to multiple protocols applied within study period. Thus, neglecting effects of chemotherapy in an older aged cohort may not possible though data shows no significance in overall survival/progression free survival in patients aged > 75 years. This topic has clearly to be addressed in prospective trails with regard to different protocols and treatment toxicity. Although to our best knowledge, this is the relatively large series reported for this population in ESCC, the sample size is still quite small. Well-designed prospective studies in larger sample size are needed to corroborate our results.

## Conclusions

In conclusion, the present study suggested that CRT for ESCC in patients aged 65 years or older had a significant benefit over RT alone in terms of OS and PFS. However, for patients aged 75 years or older, there was no statistically significant difference between CRT group and RT alone group. CRT should be performed with special attention in patients aged 75 years or older. High-level prospective clinical trials are needed, thus to offer a scientific basis for clinical therapy for elderly patients with locally advanced ESCC.

## Data Availability

The data that support the findings of this study are available from the corresponding author upon reasonable request.

## Abbreviations

ESCC: Esophageal squamous cell carcinoma
CRT: Chemoradiotherapy
RT: Radiotherapy
OS: Overall survival
PFS: Progression-free survival
EC: Esophageal carcinoma
dCRT: Definitive chemoradiotherapy
TNM: Tumor-node-metastasis
CT: Computed tomography
PET-CT: Positron emission tomography-CT
IMRT: Intensity modulated radiotherapy
GTV: Gross tumor volume
CTV: Clinical target volume
PTV: Planned target volume
RTOG: Radiation Therapy Oncology Group
EORTC: European Organization for Research and Treatment of Cancer radiation morbidity score system
PNI: Prognostic nutrition index
NLR: Neutrophils-lymphocytes ratio
PLR: Platelets-lymphocytes ratio
LMR: Lymphocytes-monocytes ratio
SII: Systemic immune-inflammation index
HR: Hazard ratio
CI: Confidence interval
OR: Odds ratio
RE: Radiation esophagitis
RP: Radiation pneumonitis
